# Relation of cholesterol metabolism to pediatric gallstone disease: a retrospective controlled study

**DOI:** 10.1186/s12876-015-0304-4

**Published:** 2015-06-30

**Authors:** Antti Koivusalo, Mikko Pakarinen, Helena Gylling, Markku J. Nissinen

**Affiliations:** 1 Hospital for Children and Adolescents, University of Helsinki and Helsinki University Central Hospital, Helsinki, Finland; 2 Department of Medicine, Division of Internal Medicine, University of Helsinki and Helsinki University Central Hospital, Helsinki, Finland; 3 Department of Medicine, Division of Gastroenterology, University of Helsinki and Helsinki University Central Hospital, Helsinki, Finland; 4 Biomedicum Helsinki, Room C422, POB 700, FI-00029 HUS, Helsinki, Finland

**Keywords:** Black pigment gallstones, Cholesterol gallstones, Non-cholesterol sterols, Plant sterols

## Abstract

**Background:**

Cholesterol metabolism may be involved in pediatric gallstone disease. We aimed to reveal cholesterol metabolites and phytosterols and their relation to stone composition of sterols in children having black pigment and cholesterol stones.

**Methods:**

We performed retrospective controlled clinical study, in which we examined parameters of cholesterol metabolism and liver function values in serum (*n* = 28) and gallstones (*n* = 46) of consecutively cholecystectomized children. Serum values of age-, body mass index- and sex-matched children (*n* = 82) and adult gallstones (*n* = 187) served as controls.

**Results:**

Surrogate markers of cholesterol synthesis in serum (squalene/cholesterol, cholestenol/cholesterol and lathosterol/cholesterol) were 26–52 % higher in both stone subclasses compared to controls (*p* < 0.05 for all). Respectively, cholestanol/cholesterol and plant sterols campesterol/cholesterol and sitosterol/cholesterol (cholesterol absorption markers) had decreasing order in serum: black pigment stone group > controls > cholesterol stone group (*p* < 0.05 for all). In black pigment stone group, stone cholestanol/cholesterol was associated with serum bile acids (*r* = 0.620, *p* = 0.018). In cholesterol stone group, surrogate markers of cholesterol synthesis in serum (*e.g.,* lathosterol/cholesterol) inversely reflected those of absorption (*r*-range -0.633–-0.706, *p*-range 0.036–0.015). In cholesterol stone group, serum and stone lathosterol/cholesterol and cholestanol/cholesterol were positively interrelated (*r*-range 0.727–0.847, *p* < 0.05 for both).

**Conclusions:**

Gallstone subclasses shared enhanced cholesterol synthesis. Cholesterol stone children were low cholesterol absorbers with intact homeostasis of cholesterol metabolism. Black pigment stone group was characterized by deteriorated cholesterol metabolism, and accumulation of cholestanol, campesterol and sitosterol in serum and stones suggesting their participation in pathogenesis.

## Background

Gallstones are quite rare in healthy children, but they more frequently occur in subjects who have a predisposing condition [1, 2]. Recent series document an increasing incidence of pediatric gallstone disease most probably due to increased detection with frequent use of ultrasonography, as well as the growing obesity epidemic [3]. Grading of gallstones into black pigment (BPS), brown pigment (BRPS) and cholesterol stones (CS) can be performed by determining the stone cholesterol content [4–7]. More exact classification methods apply ultrastructural examination with scanning electron microscopy, x-ray diffractometry and infrared spectroscopy [8].

Numerous conditions have been associated with the pathogenesis of BPS, in which the elevated biliary bilirubin level is the cornerstone phenomenon, and this is frequently a consequence of chronic haemolysis or hepatobiliary disease [2, 6]. In turn, BRPS have been related to parenteral nutrition even more tightly than the BPS [7]. CS, which are more frequent in older children and adults than pigment stones (PS), are formed by several pathophysiologic factors, of which certain properties in cholesterol metabolism play essential roles. These metabolic features include hepatic hypersecretion of biliary lipids, enhanced phase transition of cholesterol, high efficiency of intestinal cholesterol absorption and increased hepatic biosynthesis of cholesterol [9].

Cholesterol homeostasis is maintained by the interaction between intestinal absorption, *de novo* synthesis, hepatic output into the bile and fecal secretion of cholesterol [10]. Phytosterols are not synthesized in the human body, but they are normally absorbed to a much lesser extent than cholesterol, and, consequently, their serum levels are only 0.1–0.005 % of total cholesterol concentration [11]. Of phytosterols in serum, campesterol and sitosterol are the most abundant ones, whereas stigmasterol and avenasterol form minor fractions. That ratios to cholesterol of campesterol, sitosterol and cholestanol (a 5-α saturated, enzymatically formed derivative of endogenous cholesterol) in serum are associated with the efficiency of cholesterol absorption among human subjects has been documented in detail [12–17]. Generally in serum, the ratios to cholesterol of squalene, lanosterol and cholesterol demethylated precursor sterols, *i.e.,* cholestenol, desmosterol and lathosterol, positively reflect cholesterol synthesis and inversely cholesterol absorption efficiency and the respective surrogate sterol markers of cholesterol absorption [13–17]. These variables of cholesterol metabolism occur also in bile and gallstones [5, 7]. Additionally, serum level of lathosterol reflects the hepatic HMG-CoA reductase activity [17, 18].

The main focuses of the present study were addressed to the following questions: 1) is the cholesterol metabolism deteriorated in pediatric BPS and CS patients compared to each other or healthy controls and 2) how the serum levels of cholesterol and the surrogate markers of cholesterol metabolism are related to the respective sterol composition of the gallstones. Thus, we analysed variables of cholesterol metabolism in serum samples (*n* = 28) and gallstones (*n* = 46) of consecutively cholecystectomized pediatric gallstone patients of a tertiary referral centre, and compared the serum values with age-, BMI- and sex-matched controls (serum) and the gallstone values with respective adult ones.

## Methods

### Patients

Gallstones of consecutively cholecystectomized children (<19 years old) from April 2004 – to January 2013, and, respectively, serum samples from January 2009 to January 2013 were retrospectively examined for the purposes of the present study. Preliminary results of solely the analysed gallstones of 18 subjects have been reported earlier [7].

Gallstones were identified preoperatively with ultrasonography in all patients. In addition, twelve patients underwent MRI cholangiography. Preoperatively bile duct stones were detected in five patients, four of whom had removal of stones by endoscopic sphincterotomy and in one the bile duct stone passed spontaneously into the duodenum. One BPS patient and two CS patients belonged to the cohort with measured composition of sterols in the gallstones. Two BPS patients belonged the cohort with serum measurements.

The term idiopathic gallstone disease was defined as occurrence of gallstones without any associated disease. Other diseases were excluded by clinical examination, abdominal ultrasound and blood samples, which were analysed for haemoglobin, white and red blood cells, platelets, alkaline phosphatase (ALP), alanine and aspartate aminotransferases (ALT and AST, respectively), bile acids, bilirubin, conjugated bilirubin, C-reactive protein, glutamyl transferase (γGT) and sedimentation rate by routine hospital methods. Patient demographic data was obtained including age, gender, height and weight at admission. Past and present medical history was collected including associated medical illnesses and medication. The use of parenteral nutrition was recorded.

The study was performed according to the principles of the 1975 Declaration of Helsinki (6th revision, 2008). The study was approved by the Ethics Committee of the Hospital for Children and Adolescents, University of Helsinki. An informed consent was obtained from parents of each patient included in the study.

### Methods

Fasting serum samples were drawn at the time of the cholecystectomy. Concentrations of cholesterol, squalene, cholestanol, cholesterol precursor sterols (lanosterol, cholestenol, desmosterol and lathosterol), and plant sterols (campesterol, sitosterol, avenasterol and stigmasterol) in serum were measured from nonsaponifiable material by gas–liquid chromatography (GLC), on a 50-m long SE-30 capillary column (Ultra 1 column, Hewlett-Packard) as described earlier [19, 20]. Serum total, LDL and HDL cholesterol and serum triglycerides were analyzed enzymatically using automated analyzer systems.

During the cholecystectomy, gallstones were collected from the gallbladder for analysis. Gallstone sterols and squalene were determined with GLC as described in detail earlier [5, 7] and shortly reviewed here. The gallstones were washed with distilled water, dried in a desiccator and powdered with mortar. The gallstone pulver (25-100g) was saponified with 2 M KOH in 90 % ethanol for two hours using 5α-cholestane as an internal standard. After saponification, aqua was added to achieve 1:1 (vol/vol) water-ethanol suspension. The nonsaponifiable lipids were extracted with hexane, washed once with 50 % ethanol, dissolved twice with hexan-methanol (2:3 vol/vol), evaporated to dryness and finally silylated to trimethylsilylethers and quantitated by GLC.

Serum levels of bile acids (available in 14 BPS and 10 CS patients), bilirubin, liver enzymes and thromboplastin time were determined by routine hospital methods prior to cholecystectomy.

### Calculations

Classification of the gallstones into CS, BRPS and BPS subclasses was performed as described earlier [5, 7]. Stones containing cholesterol less than 35 % of the stone weight were classified as PS, and greater than 35 % as CS. Based on the cholesterol content, PS were subdivided into BRPS (containing cholesterol 10 to 35 % of the stone weight) and BPS (correspondingly less than 10 %). Practically the BRPS subgroup, characterized by calcium palmitate, included mixed cholesterol gallstones and the BPS included the so-called calcium carbonate stones [8, 21–23].

The results of three patients with BRPS were published in our preliminary report [7], and, thus, the BRPS group was excluded from the present study. The results of the previously analysed and preliminarily reported [7] gallstones of 18 children were combined with respective values of 28 subjects, who participated the study from January 2009 onwards, because the composition of gallstone sterols in these two pediatric cohorts were comparable.

To eliminate the effect of varying lipoprotein levels, serum non-cholesterol sterol values were related to serum cholesterol and are expressed in terms of 10^2^ × mmol/mol of cholesterol of the same GLC run (ratios). Ratios of surrogate sterol markers of cholesterol synthesis (*e.g.*, lathosterol) to those of cholesterol absorption (*e.g.*, campesterol) in serum were calculated in order to evaluate differences in cholesterol metabolism.

The serum variables were compared to an age-, sex- and BMI- matched control group of 82 subjects, which consisted of healthy day-case surgery patients (*i.e.*, inguinal hernia, umbilical hernia, undescended testes) without evidence of intestinal disease, diabetes, or dyslipidemia. The Finnish reference values –based body mass index-for-age (ISO-BMI) were used for children over two years old [24].

The gallstone variables were compared to the respective adult values including 178 cholesterol stones (ACS) and 9 black pigment stones (APS) [5]. In this study, the term “adult controls” refers to gallstone composition of cholesterol and non-cholesterol sterols.

In order to standardize the variations, the stone non-cholesterol sterols were expressed as milligrams by stone weight and in terms of 10^2^ × mmol of non-cholesterol sterol /mol of cholesterol (called proportions or ratios in the text).

### Statistical analyses

The data were analysed for significance and normality with the Number Crunching Statistical Software™ (NCSS™, Statistical Solutions Ltd., 2007, Kaysville, Utah). Logarithmic transforms were calculated for skewed distributions. Comparisons between gallstone subclasses and controls were performed with general linear model analysis of variance using age, ISO-BMIs and gender as covariates. If *p*-values were below 0.05, comparison between the groups were carried out with two-tailed unpaired *t*-test. Categorical variables were summarized as count and compared using the chi-squared or Fisher’s exact test where appropriate. Correlations were analysed by calculating Pearson’s correlation test or by Spearman rank correlation test in case of skewed distributions. The sample sizes for the study groups were calculated according to the following hypotheses. Pediatric gallstone sterol compositions differ by several fold between the pediatric BPS and CS patients [7]. Thus, it could be estimated that the difference in serum non-cholesterol sterol ratios (*e.g.*, lathosterol/cholesterol and campesterol/cholesterol) is two-fold or more between BPS and CS patients, and that the differences from the respective control values is 40 % or more. The type I error rate and the power of the study were considered to be 5 % and 80 %, respectively. Consequently, at least 8 patients in a subgroup were required for the study using a two-sided test. A *p*-value <0.05 was considered significant.

## Results

### Characteristics of the patients and the gallstones

**Fig. 1:**
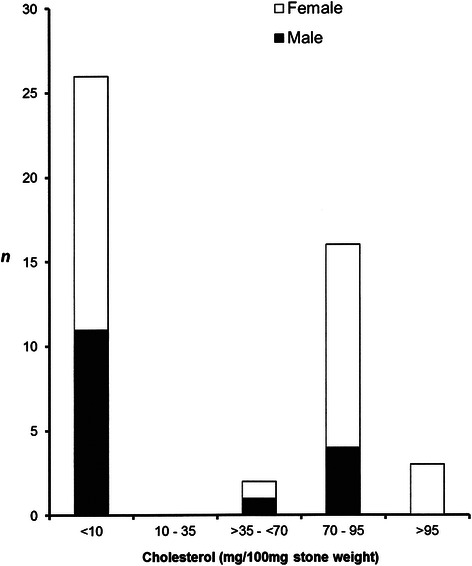
Columns show distribution of 46 cholecystectomized subjects according to gender and stone cholesterol content. Brown pigment stones (cholesterol content 10–35 mg/100 mg stone weight) were excluded in the present study

The whole study group consisted of 46 consecutively cholecystectomized children (median age 13.5 years; range 0.2–18.9; median ISO-BMI 21.5 kg/m^2^; range 14.4–31.0), of whom 31 were female (Table 1, Fig. 1). All the patients had symptoms related to gallstone disease.

**Table 1:** Characteristics of the study patients in whole study group (*n* = 46)^1^ and in a subgroup, in which both serum and gallstones values were analysed (*n* = 28)

Parameter	BPS	CS	Controls	*P*
Subgroup: serum and gallstones analysed (*n* = 28)				
Subjects *n* (%) / female *n* (%)	17(61)/9(53)	11(39)/8(73)	82/37(45)	0.215
Age (years)	10.5 ± 1.6	14.1 ± 2.0	10.7 ± 0.7	0.198
ISO-BMI (kg/m2)	21.3 ± 1.2	25.2 ± 1.4	22.5 ± 0.7	0.085
Whole group: gallstones analysed only (*n* = 46)				
Subjects *n* (%) / female *n* (%)	26(57)/15(58)	20(43)/16(80)	187/138(74)	0.386
Age (years)	11.2 ± 0.9*	14.1 ± 0.6	Adults	0.02
ISO-BMI (kg/m2)	20.9 ± 0.9*	25.7 ± 1.1	Adults	0.001

Values are means ± SEM

*BPS*, black pigment stones, *CS* cholesterol stones, *ISO-BMI* age- and sex- matched body mass index

^1^Preliminary results of gallstone analysis of 18 subjects have been reported earlier [7]

BPS were more abundant in children than in adults (57 % versus 5 %, *p* < 0.001). The 26 patients with BPS (median age 11.9 years; range 0.2–18.9; median ISO-BMI 19.8 kg/m^2^; range 14.4–25.5; 15 girls) were on an average 3 years younger than the 20 CS ones (16 girls), and, respectively, the median of their ISO-BMI was 26 % lower (*p* < 0.05 for both) (Table 1). However, among those children, who participated in serum analysis (*n* = 28, 17 girls), gender distribution, age and ISO-BMI were equal between the stone subclasses and the controls (Table 1). Analysis of covariance showed that these confounding factors did not change the statistically significant differences in serum and gallstone values between the groups reported here. Two patients were Africans, the other BPS ones were Caucasians.

In the CS-group, most of the gallstones were idiopathic (70 %), whereas in the BPS-group the etiopathogenesis was more heterogeneous, and the most common clinical diagnosis were haemolytic diseases (38 %) and idiopathic (27 %) (Table 2). Only four PS patients had a history of parenteral nutrition. Preoperative serum values of haemoglobin, white and red blood cells, platelets, C-reactive protein and sedimentation rate were within normal limits in both subgroups of patients (not shown). Preoperative serum levels of γGT were elevated by 1.6- - 5-fold in 19 % of the patients with BPS and in 10 % among those with CS (*p* = 0.33) (Table 3). The median of total bilirubin level in serum was two times higher in BPS than in CS (*p* = 0.02) (Table 3). Respectively, serum levels of ALP were above the upper limit of normal by 1.1- - 1.3-fold in 8 % (BPS-group) and in 9 % (CS-group) (*p* = 1.00).

**Table 2:** Past or present clinical diagnosis in gallstone patients

Diagnosis	All	BPS	CS
	*n* = 46	*n* = 26	*n* = 20
Idiopathic gallstones	21 (46)	7 (27)	14 (70)
Hemolytic disease	11 (24)	10 (38)	1 (5)
Neonatal hyperbilirubinemia^1^	5 (11)	5 (20)	-
History of parenteral nutrition	5 (11)	5 (19)	-
Inflammatory bowel disease	3 (7)	3 (12)	-
Epilepsy with medication	2 (4)	-	2 (10)
Extrahepatic portal vein occlusion	2 (4)	1 (4)	1 (5)
Transplanted kidney	2 (4)	2 (8)	-
Acute leukemia	1 (2)	1 (4)	-
Intestinal failure, transplanted small bowel	1 (2)	1 (4)	-
Meningomyelocele	1 (2)	-	1 (5)
Neurofibromatosis	1 (2)	-	1 (5)
Octreotide treatment, pancreatitis (non biliary)	1 (2)	1 (4)	-

Values are *n* (%)

*BPS* black pigment stones, *CS* cholesterol stones

^1^neonatal hyperbilirubinemia is included in the count of hemolytic disease

**Table 3:** Preoperative liver biochemistry of gallstone patients

	BPS	CS	*p* value^b^
Patients (*n*)	26	20	
Plasma aspartate aminotransferase (IU/L)	32 (9–122)	22 (17–67)	0.07
*n* (%)^a^	2 (8)	2 (10)	1.00
Plasma alanine aminotransferase (IU/L)	22 (12–26)	17 (10–306)	0.43
*n* (%)^a^	3 (12)	3 (15)	1.00
Plasma glutamyl transferase (IU/L)	25 (9–55)	19 (13–51)	0.33
*n* (%)^a^	5 (19)	2 (10)	0.45
Plasma bilirubin (μmol/L)	11 (6–187)	5 (4–52)	0.02
*n* (%)^a^	10 (38)	2 (10)	0.04
Plasma bilirubin, conjugated (μmol/L)	4 (1–10)	2 (1–13)	0.06
*n* (%)^a^	2 (8)	1 (5)	1.00
Serum bile acids (μmol/L)	8 (2–32)^c^	4 (2–151)^d^	0.75
*n* (%)^a^	7 (50)	1 (10)	0.08

Data are median (range)

*BPS* black pigment stones, *CS* cholesterol stones

^a^Number of patients (percentage) off normal range

^b^Comparison between patient subgroups using Fisher’s exact test or Mann Whitney *U*-test

^c^Data available in 14 patients

^d^Data available in 10 patients

Of the BPS, 23 % were single, 8 % were multiple and 69 % were microlithiasis. Respective percent values for CS were 20 %, 25 % and 55 % (*p* = 0.267).

Of the five patients with bile duct stones, three had BPS and two had CS. These children had no specific characteristics in their serum sterol levels or in the composition of sterols in the gallstones.

One CS patient was African, one was American Indian, but the other CS ones were Caucasians.

### Serum concentrations of enzymatic cholesterol, lipoprotein lipids and bile acids

The mean serum concentrations of lipoprotein lipids and bile acids were equal between the BPS and CS subclasses. The respective concentrations (±SEM) were for enzymatic cholesterol: 3.4 ± 0.3 mmol/l and 4.1 ± 0.3 mmol/l, for LDL-cholesterol 2.0 ± 0.2 mmol/l and 2.6 ± 0.3 mmol/l, for HDL-cholesterol 1.2 ± 0.1 mmol/l and 1.4 ± 0.1 mmol/l, for triglycerides 1.2 ± 0.2 mmol/l and 1.1 ± 0.2 mmol/l and for bile acids 10 ± 8 μmol/l and 21 ± 10 μmol/l.

### Serum levels of squalene, cholesterol and non-cholesterol sterols

Compared to the control group, the BPS subclass was characterized by 20–106 % higher ratios to cholesterol of squalene, cholestenol, lathosterol and desmosterol (*p* < 0.05 for all) (Table 4). Respectively, that of cholestanol was ~11 % higher, but stigmasterol ~41 % lower than control values (*p* < 0.05 for both).

**Table 4:** Comparison of serum cholesterol, squalene and non-cholesterol sterols in patients with gallstones and controls

	BPS	CS	Controls	*P*
	(*n* = 17)	(*n* = 11)	(*n* = 82)	
Cholesterol	129 ± 9^a^	158 ± 9	145 ± 3	*0.042*
Squalene^1^	23 ± 2^b^	28 ± 3^c^	17 ± 1	*0.003*
Lanosterol^1^	8 ± 2	11 ± 1	−^2^	*0.167*
Cholestenol^1^	31 ± 4^d^	24 ± 5^e^	15 ± 2	*0.010*
Lathosterol^1^	137 ± 17^f^	168 ± 21^g^	87 ± 8	*0.007*
Desmosterol^1^	107 ± 7^h^	104 ± 8	86 ± 3	*0.035*
Cholestanol^1^	186 ± 9^i^	126 ± 11^j^	168 ± 4	*<0.001*
Campesterol1	312 ± 34^k^	207 ± 41^l^	310 ± 15	*<0.001*
Sitosterol^1^	170 ± 18^m^	117 ± 22^n^	176 ± 8	*0.001*
Avenasterol^1^	45 ± 4	40 ± 5^o^	48 ± 2	*0.037*
Stigmasterol^1^	17 ± 3^p^	17 ± 3^q^	29 ± 1	*<0.001*
Lathosterol/Campesterol	0.58 ± 0.19^r^	1.87 ± 0.68^s^	0.34 ± 0.02	*<0.001*

Values are mean ± SEM

*BPS* black pigment stones, *CS* cholesterol stones

^1^100× mmol/mol of cholesterol

^2^Not determined

^a^*P* = 0.045 compared to CS. ^b^*P* = 0.015 and ^c^*P* < 0.001 compared to controls. ^d^*P* =0.002 and ^e^*P* = 0.009 compared to controls. ^f^*P* = 0.006 and ^g^*P* = 0.029 compared to controls. ^h^*P* = 0.003 compared to controls. ^i^*P* < 0.001 compared to CS and *P* = 0.007 compared to controls. ^j^*P* < 0.001 compared to controls. ^k^*P* = 0.002 compared to CS and ^l^*P* < 0.001 compared to controls. ^m^*P* = 0.006 compared to CS and ^n^*P* < 0.001 compared to controls. ^o^*P* = 0.014 compared to controls. ^p^*P* < 0.001 and ^q^*P* = 0.001 compared to controls. ^r^*P* = 0.010 compared to CS and ^s^*P* < 0.001 compared to controls

Compared to the control group, the CS subclass also had high ratios to cholesterol of squalene and cholesterol precursor sterols excluding desmosterol (21–93 %, *p* < 0.05 for all), but low those of cholestanol and plant sterols (17–41 % below the control values, *p* < 0.05 for all) (Table 4).

The differences between the subclasses were 18 % lower cholesterol concentration, but 31–34 % higher ratios to cholesterol of cholestanol, campesterol and sitosterol in the BPS group than in the CS one (*p* < 0.05 for all) (Table 4). The ratio to campesterol of lathosterol in the CS subclass was 3.2-fold (*p* = 0.010) and 5.5-fold (*p* < 0.001) higher than in the BPS and the control groups, respectively (Table 4).

### Gallstone sterols and squalene

Compared to the adult controls, the gallstones of the BPS-group were characterized by 2.0–3.1 –fold higher lanosterol and desmosterol values (in terms of mmol/mol of cholesterol) (Table 5). Parallel to that, also plant sterols and cholestanol values were 2.6–3.5 –fold above the controls (*p* < 0.001 for all) (Table 5).

**Table 5:** Comparison of gallstone composition of cholesterol, proportions of squalene and non-cholesterol sterols and total bile acids in black pigment stones (*n* = 26) and cholesterol stones (*n* = 20) between pediatric patients and adult controls (*n* = 187)

	Black pigment stones		Cholesterol stones		*P*
	Children	Adult controls	Children	Adult controls	
	*n* = 26	*n* = 9	*n* = 20	*n* = 178	
Cholesterol	2 ± 2^†^	3 ± 1	84 ± 2	83 ± 1	*<0.001*
Squalene	291 ± 71^†^	-	9 ± 1	-	*0.001*
Lanosterol	483 ± 21*^†^	245 ± 45	17 ± 24*	4 ± 1	*<0.001*
Cholestenol	387 ± 54	118 ± 31	378 ± 61	215 ± 8	*<0.001*
Lathosterol	956 ± 136^†^	990 ± 181	2073 ± 155	1915 ± 46	*<0.001*
Desmosterol	150 ± 769*	48 ± 12	168 ± 876*	60 ± 2	*<0.001*
Cholestanol	1233 ± 76*	480 ± 68	930 ± 87*	629 ± 22	*<0.001*
Campesterol	1046 ± 52*	311 ± 60	728 ± 59*	215 ± 8	*<0.001*
Sitosterol	1343 ± 269*^†^	389 ± 14	227 ± 307	137 ± 4	*<0.001*
Stigmasterol	209 ± 64^†^	-	16 ± 73	-	*<0.001*
Avenasterol	323 ± 31^†^	-	94 ± 36	-	*<0.001*
Bile acids	7.5 ± 1.2*^†^	3.8 ± 1.0	1.9 ± 0.5	0.7 ± 0.1	*<0.001*

Values are mean ± SEM. Cholesterol and bile acids mg/100 mg of stone. Squalene and non-cholesterol sterols 100 x mmol/mol of cholesterol

*p < 0.05 compared to the respective value in adults

^†^*p* < 0.05 compared to the cholesterol stones

The respective comparison in the CS-group revealed that lanosterol and desmosterol were 2.8–4.0 -fold above the controls (in terms of mmol/mol of cholesterol), and the relative markers of cholesterol absorption, *i.e*., cholestanol and campesterol, were 1.5–3.4 –fold higher than in controls (*p* < 0.001 for all) (Table 5).

The comparison of the two stone subclasses with each other showed that the cholesterol content in the BPS stones (mg/100 mg of stone) was, by definition, ~ 2 % of the CS-value (*p* < 0.001) (Table 5). Compared to the CS, in terms of mmol/mol of cholesterol, the BPS were rich in squalene and lanosterol, but lathosterol was only 46 % of the CS-value (*p* < 0.001 for both). Respectively, plant sterols sitosterol, stigmasterol and avenasterol, were 3–13 –fold higher in the BPS-subclass (*p* < 0.001 for all) (Table 5).

The total absolute amount of stone bile acids in BPS was double the controls and 4-times above that of CS (*p* < 0.05 for both) (Table 5).

### Correlations

The age at the time of the cholecystectomy had practically no relation to the variables studied here. ISO-BMI was inversely related to the stone cholestanol proportion in the CS group only (*n* = 20, *r* = −0.586, *p* = 0.007).

**Fig. 2:**
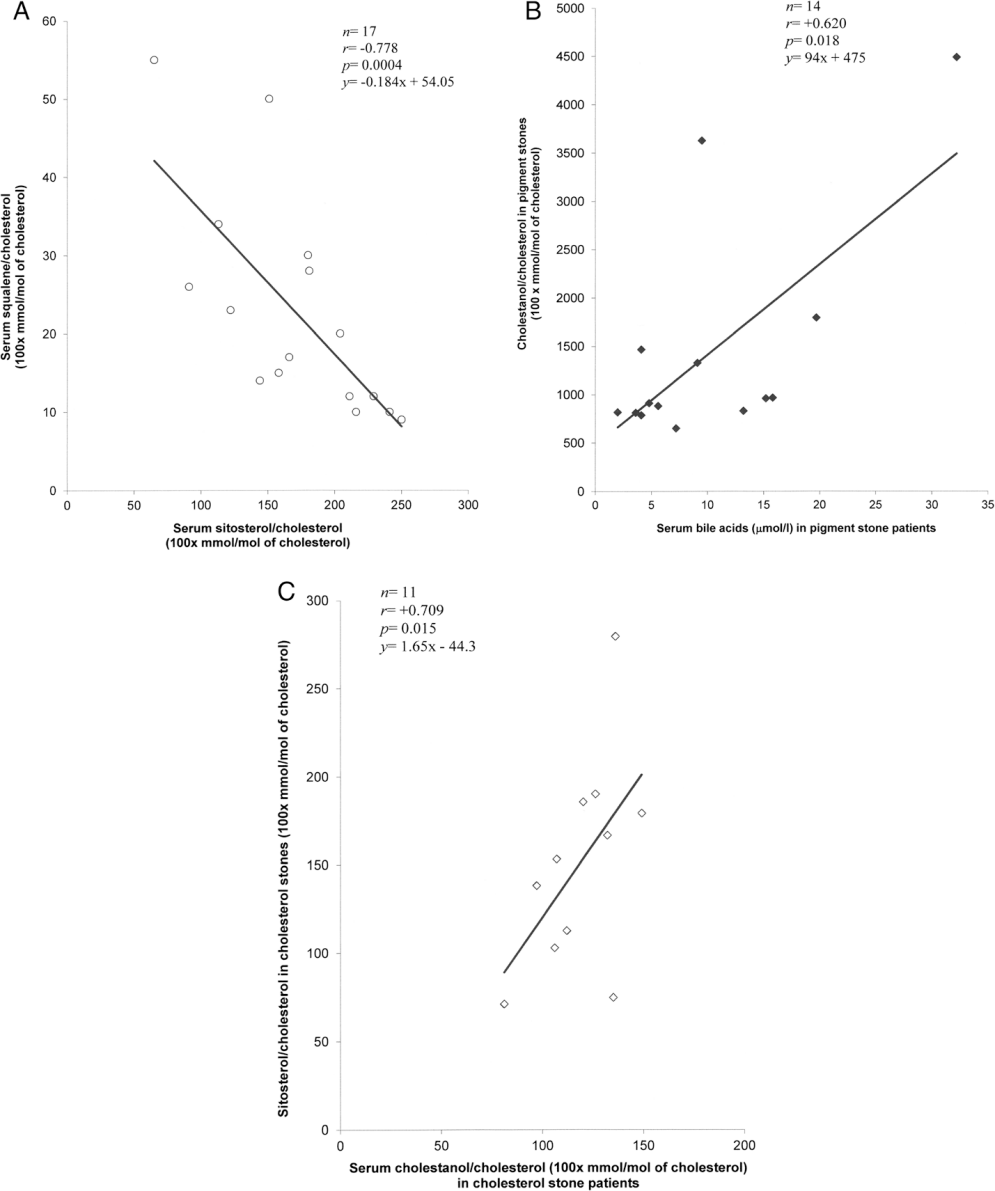
**a** Correlation between serum ratios to cholesterol of squalene and sitosterol in the cholecystectomized subjects with black pigment stones. *r* = Spearman rank correlation coefficient. **b** Correlation between stone cholestanol proportion and serum bile acids in the cholecystectomized subjects with black pigment stones. *r* = Spearman rank correlation coefficient. **c** Correlation between ratios to cholesterol of stone sitosterol and serum cholestanol in the cholesterol stone subgroup. *r* = Spearman rank correlation coefficient

In the patients with BPS, serum cholesterol and non-cholesterol sterols were poorly interrelated. However, squalene, the non-sterol precursor of cholesterol (surrogate marker of cholesterol synthesis), was inversely associated with campesterol and sitosterol in serum (as ratios to cholesterol) (*r* = −0.575 and *r* = −0.697, *p* = 0.025 and 0.004, respectively) (Fig. 2a). Furthermore, the stone cholestanol proportion was positively related to the concentration of serum bile acids (Fig. 2b).

In the patients with CS, the surrogate sterol markers of cholesterol absorption cholestanol, campesterol and sitosterol were almost consistently positively interrelated in serum and gallstones, being most evident between serum cholestanol/cholesterol –ratio and the stone proportions of the three surrogate markers of absorption (*r*-range: +0.673–+0.727, *p* < 0.03 for each) (Fig. 2c). Serum cholestanol/cholesterol ratio reflected also those of campesterol and sitosterol in serum. Lathosterol/cholesterol –ratio was inversely related to that of cholestanol in serum (*r* = −0.633, *p* = 0.036). Furthermore, ratios to cholesterol of serum and gallstone lathosterol were positively interrelated (*r* = +0.847, *p* = 0.001).

## Discussion

The main new results of the present study were that: (I) BPS and CS subgroups had enhanced cholesterol synthesis, (II) in the BPS group, solely serum squalene of the surrogate markers of cholesterol synthesis was logically (inversely) related to those of cholesterol absorption, (III) the children with CS had low absorption of cholesterol, (IV) their homeostatic regulation of cholesterol metabolism was intact and (V), in the CS group, serum non-cholesterol sterols reflected their own proportions in the stone content.

Hepatic hypersecretion of biliary cholesterol with formation of cholesterol crystals from cholesterol supersaturated bile is considered to be the crucial phenomenon in the pathogenesis of the CS [6]. In general, adult gallstone patients (predominantly with CS) have high synthesis of cholesterol parallel to increased biliary output of cholesterol, but relatively low intestinal cholesterol absorption, indicating enhanced whole-body sterol clearance [25]. Opposite to the clinical picture of the CS patients, the subjects with BPS are frequently associated with chronic haemolytic disorders, in which increased biliary excretion of bilirubin may lead to calcium bilirubinate precipitation serving a nucleation core for BPS [26]. Previous data indicate that certain variants of the gene encoding the hepatobiliary sterol hemi-transporter ABCG8 and the Gilbert syndrome-associated UGT1A1 variant are related to increased risk of gallstones suggesting that increased biliary secretions of cholesterol and bilirubin are crucial in the stone pathogenesis [27–29]. The results of the present study showed that the proportions of the serum surrogate markers of cholesterol synthesis were consistently elevated in the children with BPS and CS compared to the control values. These findings are parallel to that reported in a large series of adult gallstone patients, in whom cholesterol synthesis seemed to be equal between CS and BPS groups [5]. Altogether these data indicate that both stone subclasses in children are characterized by enhanced synthesis of cholesterol and, consequently, increased biliary secretion of cholesterol, thus, sharing a common etiopathogenetic factor in the stone formation. The results of the present study also indicate, that CS children with high surrogate markers of cholesterol synthesis have low those of cholesterol absorption, and that these variables are logically inversely related to each other. Furthermore, in the CS group, the higher the ISO-BMI the lower was the serum cholestanol ratio, and, thus, intestinal cholesterol absorption. These findings suggest, first, that cholesterol metabolism in the children with CS resembles that of adult gallstone patients, secondly, homeostasis of cholesterol metabolism is undisturbed among children with CS and, thirdly, overweight together with low intestinal cholesterol absorption are associated with risk of CS in children equally to that described in adults [25]. Interestingly, studies with experimental animals suggest that in most CS-prone humans, the small intestine is flooded continuously with an abundance of liver-secreted cholesterol molecules via bile [30]. Opposite to that found among the CS-subjects, in the BPS group, of the relative cholesterol synthesis marker, solely the early precursor of cholesterol, squalene, inversely reflected those of absorption suggesting that in the BPS group normal cholesterol homeostasis was deteriorated. Comparison between the pediatric BPS and CS groups could be strengthened by expanding the number of patients in these subgroups.

The results of our previous and the present study support the view that solely the history of parenteral nutrition does not explain high proportions of campesterol and sitosterol in BPS [7]. Interestingly, high cholesterol synthesis is associated with increased biliary cholesterol secretion paralleling also increased biliary secretion of sitosterol and campesterol [31]. In the BPS group, the serum proportions of the two most abundant plant sterols – campesterol and sitosterol – were equal to those of controls supporting the view, that, first, intestinal cholesterol absorption was normal in the BPS group, and, secondly, the high proportions of plant sterols in the stones of the BPS group could at least partly be explained by increased biliary secretion of cholesterol.

Plant sterols, especially stigmasterol, have been shown to antagonize nuclear farnesoid receptor X -mediated bile acid homeostasis in hepatocytes and associate with hepatocyte damage [32, 33]. From this point of view, it was interesting to note in the present study that serum stigmasterol/cholesterol was even below that of controls in both stone subclasses making it unobvious that serum stigmasterol interferes with stone pathogenesis. However, stone stigmasterol proportion in the BPS group was high compared to that of CS putatively due to its high biliary secretion. Overall, high proportions of phytosterols (campesterol, sitosterol, avenasterol and stigmasterol) in the stones of the BPS group suggest that, first, the gallstone disease with respect to BPS differs from that of adults, and, secondly, phytosterols may be related to the formation of pediatric BPS.

The present study indicated that the children with BPS were characterized by high serum and stone proportions of cholestanol. Cholestanol is a noncholesterol sterol metabolite of cholesterol that serves as a surrogate marker of cholesterol absorption under normal physiological conditions and reflects sensitively cholestasis in primary biliary cirrhosis and biliary atresia [34, 35]. Paralleling these findings, in BPS patients of the present study, serum bile acid concentration and stone composition of cholestanol (but not plant sterols) were positively interrelated suggesting that high cholestanol proportions in the stones of the BPS group could partly be explained by cholestasis. Cholesterol precursors have been associated with some disorders in human subjects, such as squalene in cardiovascular diseases, desmosterol in steatohepatitis and 7-dehydrocholesterol in Smith-Lemli-Opitz syndrome [36–38]. In addition, subjects affected by autosomally inherited disease phytosterolemia are characterized by very high levels of phytosterols in serum and tissues [39]. So far, serum cholestanol and phytosterols have not been related to the gallstone disease in the absence of parenteral nutrition. In the present study, several subjects in the BPS group had a disease associated with haemolysis, but haemolysis itself had no influence on serum cholestanol or plant sterol proportions. Overall, high stone and serum proportions cholestanol and plant sterols together with deteriorated cholesterol homeostasis (as evaluated by the non-cholesterol sterol surrogate markers) support the views that, first, BPS patients have had at least in their history a high biliary secretion of these sterols, and, secondly, some so far unidentified factors may lie behind accumulation of these non-cholesterol sterols in the stones and serum of the BPS patients. In the pathogenesis of BPS, also several factors in the gallbladder mucosa play crucial roles [40, 41].

## Conclusions

In conclusion, the gallstones of the pediatric BPS group are characterized by high proportions of plant sterols and cholestanol, of which solely the latter one reflects cholestasis. This findings support the view that plant sterols may be involved in the pathogenesis of BPS. Further research is needed to evaluate whether screening for high serum cholestanol ratios could be helpful in early detection of gallstones at high risk groups. Cholesterol homeostasis with increased cholesterol synthesis and low cholesterol absorption together with high ISO-BMI in the pediatric CS group resembles those risk factors associated with CS disease described in adults and with the metabolic syndrome. Identification of this clinical connection gives tools to decrease the risk for the pediatric CS disease. High cholesterol synthesis in both stone subclasses support the view of increased biliary cholesterol secretion, which consequently enables cholesterol supersaturation in bile. This phenomenon displays that these two stone subclasses share a similarity with this respect in the pathogenesis of gallstone formation.

## Abbreviations

ACS: Adult cholesterol stones
APS: Adult pigment stones
BPS: Black pigment stones
BRPS: Brown pigment stones
CS: Cholesterol stones
GLC: Gas–liquid chromatography
ISO-BMI: the Finnish reference values –based body mass index-for-age
